# Apoptotic vesicles in cancer: research progress in physiology and therapy

**DOI:** 10.1186/s12964-026-02731-4

**Published:** 2026-02-27

**Authors:** Jingyu Dai, Yibing Liu, Tao Tang, Mingsheng Tang, Jun Chen, Hailan Wang, Xi Liu

**Affiliations:** 1 Department of General Surgery, Longgang Central Hospital of Shenzhen, No. 6082, Longgang Avenue, Longgang District, Shenzhen, Guangdong 518116 China; 2 Department of Endocrinology, Longgang Central Hospital of Shenzhen, No. 6082, Longgang Avenue, Longgang District, Shenzhen, Guangdong 518116 China; 3 Department of Nephrology, Longgang Central Hospital of Shenzhen, No. 6082, Longgang Avenue, Longgang District, Shenzhen, Guangdong 518116 China

**Keywords:** Apoptotic vesicles, Tumor microenvironment, Immune evasion, Drug delivery, Tumor diagnostics, Tumor therapy

## Abstract

Apoptotic cells release a highly heterogeneous population of extracellular vesicles, which can be categorized into apoptotic bodies, apoptotic microvesicles, and apoptotic exosomes. These apoptotic vesicles (apoVs) not only inherit materials from their parental cells but also encapsulate apoptosis-related factors, thereby playing multifaceted roles in signal transduction, homeostatic regulation, and the tumor microenvironment. This review highlights recent advances in the cell biology of apoptotic extracellular vesicles, with a focus on the unique characteristics of tumor-derived ApoVs and their mechanisms of interaction in immune evasion, angiogenesis, and the modulation of the tumor microenvironment. Additionally, the potential of ApoEVs as biomarkers for tumor diagnostics is explored. In the realm of tumor therapy, engineered and modified ApoEVs have emerged as promising drug delivery vehicles and vaccine carriers, enabling targeted delivery of therapeutic agents and showing significant potential in antitumor immunization strategies.

## Highlights

Summarizes the role of apoptotic extracellular vesicles in the tumor microenvironment and their clinical applications.

Evaluates the potential of these vesicles for use in tumor diagnosis and emerging therapies.

Outlines future research directions and discusses challenges for clinical translation.

**Graphical Abstract:**

## Graphical abstract

**Figure:**
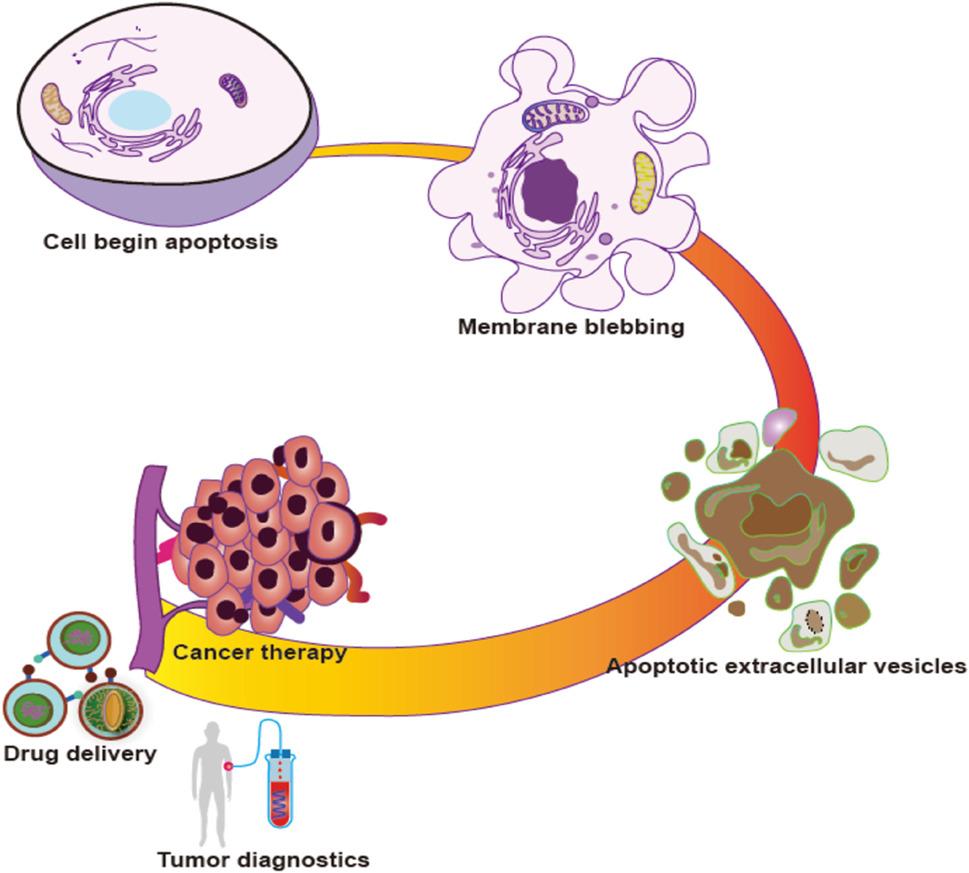
Apoptotic extracellular vesicle for advanced drug delivery and cancer therapy.

## Introduction

Over the past decade, extracellular vesicle (EV) has rapidly evolved into a cutting-edge discipline for disease treatment, with particular significance in tumor microenvironment (TME) studies. EVs are heterogeneous, lipid-bilayer-encapsulated nanoparticles that serve as key mediators of intercellular communication. By transporting bioactive molecules, including proteins, nucleic acids, and lipids, they can dynamically regulate tumorigenesis, metastasis, drug resistance, and immune escape [1].

According to the MISEV2023 guidelines [2], EVs released via the multivesicular body (MVB) from the intracellular endosomal system are designated as exosomes (30–150 nm), whereas those generated through plasma membrane budding are termed ectosomes(50–1000 nm) [3]. Furthermore, additional nomenclature is employed to categorize EVs originating from specific cellular processes such as migrasomes, which are associated with cell migration, and apoptotic extracellular vesicles (ApoEVs; 100–5000 nm), generated during programmed cell death [4]. Recent studies have identified novel subtypes such as migrasomes [5], retractosomes [6], exomeres [7], and mitochondrial-derived vesicles (mitophers) [8], outer membrane vesicles [9], further expanding our understanding of EV heterogeneity.

In oncology, therapeutic stressors (e.g., radiotherapy, chemotherapy) and TME hypoxia induce programmed tumor cell death, generating abundant tumor-derived apoptotic vesicles (apoVs) [10]. Unlike exosomes and microvesicles, apoVs as apoptotic cell disintegration products, exhibit unique molecular characteristics: broader size distribution (100-5,000 nm), enrichment of specific surface markers (phosphatidylserine, C1q complement protein, Fas death receptor), and cargo containing parental cell-derived nucleic acids, metabolites, and organelle fragments [11]. Notably, ApoEV biogenesis depends critically on caspase activity, particularly caspase-3-mediated nucleocytoplasmic translocation of key molecules (e.g., MTA1) and vesicle packaging. These properties establish ApoEVs as unique carriers of tumor biological information that regulate cancer progression through surface ligand-receptor interactions or endocytic delivery of bioactive substances.

Although exosomes and microvesicles have been extensively studied in the TME, the roles of ApoEVs remain less explored [12]. Tumor-derived ApoEVs promote malignant progression through multiple mechanisms such as metastasis, drug resistance, and immune escape. Paradoxically, while predominantly pro-tumorigenic, certain ApoEV sources exhibit intrinsic anti-tumor potential, including cancer cell apoptosis induction. As natural antigen reservoirs, tumor-derived ApoEVs carry tumor-specific antigens and danger signals, offering novel immunotherapeutic opportunities. Engineered ApoEVs can be loaded with immunostimulatory molecules to activate innate immunity and enhance anti-tumor responses. Critically, ApoEVs demonstrate advantages over live cells or other vesicles such as higher yield, superior tissue targeting, adaptable drug-loading capacity, and avoidance of tumorigenic risks and ethical concerns associated with cell therapies. Given their dual role as both drivers of cancer evolution and vectors for therapeutic innovation, this review focuses on: (1) the biogenesis and physiological properties of tumor-derived ApoEVs; (2) their mechanisms in tumor regulation; and (3) their potential in overcoming therapeutic resistance and developing targeted delivery systems. Advances in isolation techniques and engineering strategies position ApoEVs as emerging tools for precision cancer therapy are also featured in this article. Specifically, our work distinctively focuses on their dual potential as diagnostic biomarkers and therapeutic delivery vehicles within integrated discussion. Our novelty lies in systematically framing this discussion around the latest MISEV2023 guidelines, thereby providing a contemporary, methodology-forward perspective that outlines a standardized path for future ApoEV research from characterization to application (Table 1).

**Table 1:** Comparison of ApoVs with other EVs

	Exosomes	Microvesicles	Oncosomes	Migrasomes	Rafeesome-*R*-EVs	ApoVs
Physical properties	Particle diameter:30–150 nm; Buoyant density:1.08–1.14 g/ml	Particle diameter: 50–1000 nm; Buoyant density:1.16–1.19 g/ml	Particle diameter: 1 ~ 10 μm Buoyant density:1.10–1.15 g/ml	Particle diameter: 500–3000 nm	Particle diameter: 100–500 nm	Particle diameter: 100–5000 nm Buoyant density: 1.16–1.28 g/ml
Molecular markers	CD63, CD81, CD9, Flotillin, Rab, chaperones TSG101, ceramide, and Alix	Annexin A1, CD40,CD62,integrin	LO, cytokeratin 18 (CK18)	TSPAN4, TSPAN7, NDST1, EOGT, PIGK, CPQ	STING, TMEM33, RTN4	CD9/CD63/CD81 + PS+/C1q+/Fas+ C1q, PS, Fas, Intergrin alpha-5, Caveolin-1,Syntaxin-4, Cavin1
Contents	Proteins MHCI and MHCII Lipid rafts Targeting and adhesion proteins mRNAs miRNAs lncRNAs circRNAs	Cytosolic and plasma membrane proteins MHCI and MHCII Lipid rafts Targeting and adhesion proteins mRNAs miRNAs circRNAs lncRNAs	miRNAs, metalloproteinases, and Cav-1	mRNA, miRNA, damaged mitochondria, signaling molecules such as chemokines, cytokines and growth factors	Endoplasmic reticulum proteins, cytoplasmic proteins	Cytosol proteins, histones, nucleus, fragmented genomic DNA, organelles, lipids, metabolites,
Regulation of production	Endosomal pathway	Plasma Membrane Blebbing	Shedding of membrane blebs	Vesicles formed on retraction fibers of migrating cells	Non-canonical autophagosome fused with early endosome	Caspase-dependent, ROCK1-mediated contraction of actinomyosin, Form in plasma membrane, multivesicular bodies or apoptopodia
Biological function	cell–cell communication, drug delivery	cell–cell communication, package active cargo	extracellular communication	Mediates migratory cytokinesis, cell homeostasis maintenance, intercellular material transfer, intercellular signal integration	Regulation of intercellular transfer of activated STING and activated STING-containing ER induces antitumor immunity in recipient cells.	Mediates cell burial and immune recognition, Enables intercellular communication and information transfer, Tightly coupled to apoptotic processes

### Biogenesis of the apoevs

**Fig. 1:**
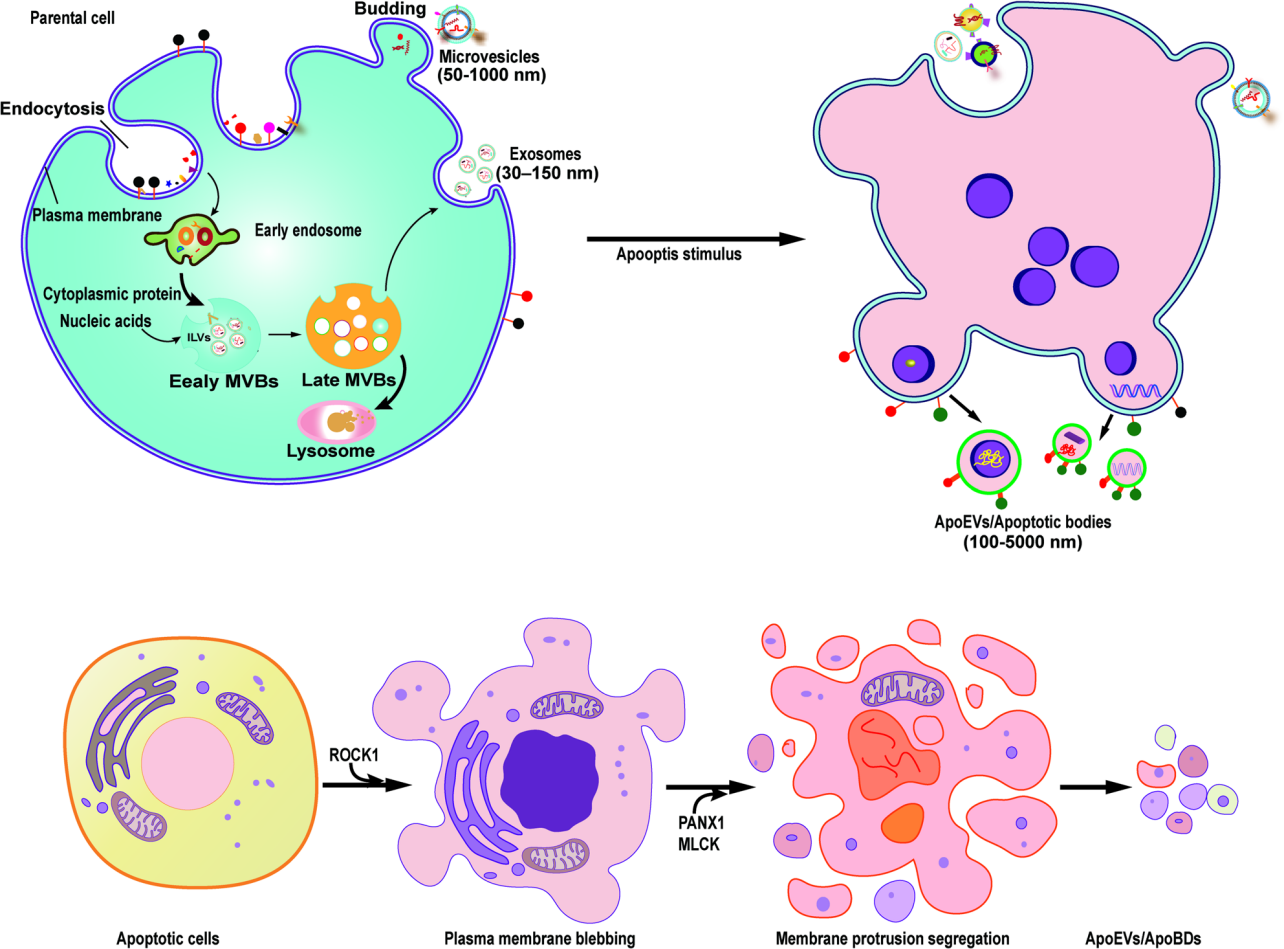
Schematically illustrates the morphology and molecular mechanisms underlying ApoEVs biogenesis.Under basal conditions (upper left), cells generate heterogeneous EV populations containing exosomes and microvesicles (MVs). However, upon apoptotic stimulation, caspase activation triggers ROCK1-mediated plasma membrane remodeling. This process subsequently induces ring-shaped bulges that evolve into distinct apoptotic structures: microtubule spikes, apoptotic vesicles, and beaded apoptotic vesicles. Ultimately, cellular fragmentation yields numerous individual ApoEVs ranging 1-5 μm in diameter. ILVs, Intraluminal vesicles, MVBs, multivesicular endosomes

Apoptosis, a genetically programmed cell death process, plays crucial roles in development, immune regulation, and tissue homeostasis. Unlike necrotic cell death, this evolutionarily conserved mechanism enables multicellular organisms to eliminate superfluous cells through controlled self-disassembly. In humans, approximately 50 billion cells undergo apoptosis per day [13]. During this process, cells exhibit characteristic morphological changes including cytoplasmic shrinkage, nuclear condensation, organelle fragmentation, and plasma membrane blebbing (Fig. 1) [14]. Ultimately, nuclear disintegration and membrane rupture generate ApoEVs, which are subsequently engulfed by phagocytes to prevent inflammatory spillover [15, 16]. Critically, apoptosis generates bioactive metabolites and apoptotic extracellular vesicles (apoEVs) that carry molecular cargo from parent cells, contributing to physiological homeostasis though their specific biological functions require further elucidation.

ApoEV biogenesis represents the terminal phase of programmed cell death, dependent on caspase cascade activation, particularly caspase-3. Upon apoptotic stimulation, activated caspase-3 cleaves and activates Rho-associated coiled-coil kinase 1 (ROCK1), triggering actomyosin contraction that drives cytoskeletal reorganization and initial membrane blebbing [16, 17]. Concurrently, caspase-mediated inhibition of phospholipid flippases (e.g., ATP11) combined with activation of scramblases (e.g., Xkr8/TMEM16F) induces phosphatidylserine (PS) externalization [18]. This PS exposure serves not only as an apoptosis hallmark but also as an “eat-me” signal for efferocytosis [19].

ApoEVs formation progresses through three distinct morphological stages: *Initially*, ROCK1 activation induces LIM kinase 1 phosphorylation, promoting cofilin phosphorylation and subsequent actin depolymerization [20]. *Subsequently*, actomyosin contraction synergizes with microtubule dynamics to condense nuclear material, while localized hydrostatic pressure generates membrane protrusions (e.g., apoptotic feet, beaded vesicles) that encapsulate cytoplasmic components. *Ultimately*, plasma membrane separation from the actin cortex occurs under tensional equilibrium, with rapid cytoskeletal remodeling constraining vesicle expansion [21]. This process involves coordinated regulation by multiple molecular networks: the core caspase-ROCK1 axis; Bcl-2 family proteins modulating mitochondrial permeability; myosin light chain kinase (MLCK) facilitating nuclear packaging; ADP-ribose polymers promoting chromatin condensation; and Pannexin 1 (PANX1) channels acting as negative regulators by limiting membrane protrusion segregation. ApoEVs exhibit substantial heterogeneity based on size and biogenesis: Apoptotic bodies (ApoBDs, 1–5 μm) originate from membrane protrusions and may contain organelles, requiring microtubule-associated structures [22]. Apoptotic microvesicles (ApoMVs, < 1 μm) derive directly from plasma membrane budding [23, 24]. Wherease, apoptotic exosomes (ApoExos) form via sphingosine-1-phosphate receptor (S1PR) signaling and autophagosome interactions [25]. Parental cell lineage determines ApoEV composition (e.g., DNA-rich vesicles from T cells, mitochondria-enriched vesicles from monocytes), whereas apoptotic stimuli primarily modulate vesicle yield. Notably, large senescent neutrophil-derived vesicles (LAND-V) generated through RhoA-mediated budding carry surface CD55 molecules that inhibit complement C3 convertase, demonstrating subtype-specific functionality.

 In vivo, ApoEVs display organotropic distribution (skin-enriched, liver-cleared) and mediate critical physiological functions. During liver regeneration, for instance, hepatocyte-derived ApoEVs are internalized by neutrophils, inducing a pro-regenerative phenotype that secretes fibroblast growth factor-2 (FGF2) to facilitate tissue repair [26]. These functions stem from their molecular cargo: beyond PS exposure, ApoEVs transport apoptotic signaling molecules (C1q, Fas ligand), nucleic acids (e.g., miRNA-21-5p), and parent cell-derived proteins/metabolites that collectively constitute apoptotic messaging vectors.

The MISEV2023 guidelines, released by the International Society for Extracellular Vesicles, establish a systematic framework for identifying and characterizing apoptotic extracellular vesicles (ApoEVs). Although the guidelines do not contain a dedicated section on ApoEVs, their core principles remain fully applicable. Successful implementation requires careful attention to the apoptotic origin and unique molecular signatures of ApoEVs within the standard workflow. Given their clear apoptotic origin, ApoEVs can be described as “large EVs” or “apoptosis-associated EVs.” In separation and concentration, the chosen method must explicitly state whether it prioritizes high yield or high specificity, and any co-isolated impurities should be reported. For ApoEVs, differential ultracentrifugation is commonly employed for enrichment. However, caution is necessary due to potential co-precipitation with apoptotic bodies and cellular debris. Consequently, evaluating and reporting the efficiency of apoptosis induction is essential for assessing yield and purity. The characterization phase strictly requires validation through at least two orthogonal methods. In practice, nanoparticle tracking analysis (NTA) determines particle size and concentration, whereas transmission electron microscopy (TEM) visually confirms their spherical, membrane-bound morphology. Additionally, Western blot analysis is used as MISEV recommendations to detect positive EV-associated markers (e.g., CD63) and negative markers (e.g., calnexin), thereby confirming the presence of EV proteins and absence of intracellular contaminants. The detection of apoptosis-specific markers, such as externalized phosphatidylserine (typically verified by Annexin V-binding flow cytometry), also provides key identification evidence for ApoEVs. Functional assays, including studies of cellular uptake, may be conducted as needed. Throughout, rigorous reporting of particle characteristics, protein markers, and contaminant profiles is required to ensure methodological robustness and reproducibility.

### Biological and physiological characteristics of ApoVs

ApoEVs, as key products of apoptotic programming, play complex and multifaceted roles in physiological and pathological contexts, extending beyond traditional “cellular debris” concepts. In physiological homeostasis, ApoEVs primarily ensure efficient clearance of daily apoptotic debris through coordinated mechanisms: size-optimized vesicle formation enables macrophage recruitment via motility-triggered active search, while surface phosphatidylserine (PS) exposure guides phagocyte migration through chemokine signaling. This cellular clearance machinery, regulated by key molecules like PANX1 and ROCK1, maintains tissue integrity. Conversely, in disease states such as osteoarthritis, synovial fluid ApoEVs deliver miR-21-5p that upregulates MMP-13 and accelerate cartilage degradation [27], while glioma-derived ApoEVs enhance malignant phenotypes [28].

Immunologically, ApoEVs exhibit context-dependent duality. Under homeostasis, their phagocytosis induces anti-inflammatory mediator release, promotes M2 macrophage polarization, and triggers dendritic cell migration to lymph nodes where TGF-β secretion induces peripheral T-cell tolerance and regulatory T-cell differentiation, establishing immune silence. Conversely, in pathological environments (e.g., tumors or infections), ApoEVs carrying tumor-specific antigens or pathogen components undergo efficient dendritic cell cross-presentation, activating CD8⁺ T-cell adaptive immunity, thus property enabling their development as anticancer/anti-infection vaccine carriers [29]. Their advantages include dedicated phagocyte recognition, cargo protection, and efficient MHC-antigen complex formation. For instance, dendritic cells pulsed with β-cell-derived ApoEVs significantly reduce diabetes incidence in models, neutrophil ApoEVs deliver anti-inflammatory drugs to resolve post-MI inflammation, and cGAMP-loaded ApoEVs enhance antitumor immunity [23, 30–32].

Moreover, ApoEVs directly regulate cell fate and tissue regeneration [33]. Hepatocyte-derived ApoEVs activate JAK/STAT and PI3K/Akt/NF-κB cascades to enhance stellate cell survival [34]. A zebrafish models demonstrate stem cell-derived ApoEVs induce phagocytosis via Wnt8a signaling and osteoblast-derived ApoEVs promote preosteoblast differentiation through RANK-mediated PI3K/Akt/mTOR activation, highlighting regenerative potential. Hepatocyte-derived ApEVs deliver cyclin-dependent kinase 1 (Cdk1) to orchestrate liver regeneration by stimulating neighboring hepatocyte proliferation [35]. Pathologically, defective ApoEV clearance drives atherosclerotic necrotic core formation and vascular calcification, whereas post-myocardial infarction ApoEVs target hypoxic regions to inhibit inflammatory cell death and facilitate tissue repair [36].

Impaired clearance of ApoEVs triggers serious pathological consequences. Untimely cleared ApoEVs rupture, releasing nucleosomal histones, nucleic acids, damage-associated molecular patterns (DAMPs), and autoantigens that induce secondary necrosis and chronic inflammation. This process drives necrotic core formation and vascular calcification in atherosclerosis, promotes autoimmune T-cell responses through histone H3 exposure in systemic lupus erythematosus, and contributes to vitiligo pathogenesis via autoantigens such as laminin. To counteract these mechanisms, promising therapeutic strategies include use PANX1 inhibitors (e.g., probenecid) that restore efficient clearance; and engineered ApoEVs loaded with cGAMP for enhanced anti-tumor immunity, anti-inflammatory agents for myocardial repair, or β-cell antigens for diabetes prevention [23]. These advances confirm ApoEVs function not as inert cellular debris but as dynamic signaling hubs connecting apoptosis programs with tissue homeostasis regulation, providing novel biological foundations for therapeutic development.

Notably, ApoEV functionality depends critically on parental cell identity (e.g., young MSC-derived vesicles activate autophagy for senescent cell repair), microenvironmental status, and recipient cell properties. Molecular profiling reveals ApoEV enrichment in proteins regulating cell behavior, metabolism, material transport, and disease processes. Their membranes specifically display apoptosis markers phosphatidylserine (PS) and C1q alongside universal EV markers (CD9, CD63, CD81) [37–39]. Bioinformatic analyses confirm parental signature inheritance: mesenchymal stem cell (MSC)-derived ApoEVs express CD29/CD44/CD90 [40], wherase pluripotent stem cell-derived vesicles carry caspase-3-dependent SOX2 [41], and glioblastoma-derived counterparts contain the oncogenic splicing regulator RBM11 [28]. Thus, ApoEVs possess unique biomarkers and cargo distinguishing them from classical EVs, determining their targeting specificity and functional mechanisms in pathophysiological contexts.

While ApoEVs’ physical properties and molecular composition confer distinctive biological functions, establishing them as active targeting and their therapeutic translation faces challenges. Source heterogeneity and isolation limitations necessitate establishing standardized nomenclature based on size, induction method, specific cargo, and biological effects.

### Role of apoevs in cancer progression

Apoptotic pathways in cancer cell are frequently dysregulated, with anti-apoptotic protein overexpression enabling apoptotic escape—a hallmark of malignancy [42]. Consequently, anticancer therapies (chemotherapy, radiotherapy, CAR-T) primarily eliminate cancer cells through apoptosis induction [43]. Paradoxically, these treatments generate abundant tumor-derived ApoEVs in the tumor microenvironment (TME) [44]. These vesicles mediate intercellular communication that reshapes tumor heterogeneity and accelerates cancer evolution. For example, glioblastoma ApoEVs transfer splicing factor RBM11 to recipient cells, remodeling Cyclin D/MDM4 pre-mRNA splicing to drive oncogenic subtype expression, proliferation, and drug resistance [28, 45–47]. Similarly, lung adenocarcinoma (LUAD) ApoEVs deliver ALDH1A1, activating the NF-κB/SOX2 axis to induce epithelial-mesenchymal transition (EMT), stemness, and metastasis [3]. Experimental models confirm LUAD-derived ApoEVs enhance ALDH activity to activate NF-κB signaling and promote EMT, significantly increasing tumor cell self-renewal capacity, stemness, and metastatic potential. This intercellular communication mechanism reveals new therapeutic targets for lung cancer.

Simultaneously, ApoEVs remodel the TME through multiple pathways. Epithelial ovarian cancer-derived ApoEVs contain CD147, inducing matrix metalloproteinase secretion to promote angiogenesis [48, 49]. Concurrently, ApoEVs significantly influence tumor progression through immune modulation. Lymphoma-derived ApoEVs carrying tumor-associated antigens are captured by splenic CD169⁺ macrophages, subsequently inhibiting dendritic cell-mediated cytotoxic responses and establishing systemic immune tolerance [50]. Conversely, ApoEVs can enhance immune surveillance by promoting tertiary lymphoid structure formation. During vascular endothelial injury in solid tumor patients, ApoEVs containing active 20 S proteasomes signal γδT cells to migrate to injury sites, where IL-17 production facilitates tertiary lymphoid structure maturation. This process locally activates immune responses and improves tumor clearance capacity [51]. Traditionally, research on the antitumor effects of natural products has focused predominantly on their direct inhibition of cancer cell proliferation or induction of apoptosis [52]. In the context of hepatocellular carcinoma (HCC), Oroxylin A has been demonstrated to trigger the generation of abundant ApoEVs via the caspase‑3/ROCK1 pathway. These ApoEVs are enriched in glycolytic enzymes and can be internalized by macrophages, thereby activating the ROS–NLRP3 inflammasome pathway within these immune cells. Consequently, this cascade promotes macrophage polarization toward an antitumor M1‑like phenotype [53]. The elucidation of this novel antitumor mechanism provides new perspectives for developing ApoEV‑based immunocombination therapies against hepatocellular carcinoma. In contrast, mesenchymal stem cell (MSC)-derived ApoEVs directly trigger calcium influx and apoptosis in multiple myeloma cells through Fas ligands [54]. Critically, ApoEVs can treatment drug resistance. Radiotherapy-induced cervical cancer ApoEVs deliver MTA1 to activate the p-STAT1/NR2F1 axis, inducing dormancy and radioresistance [55]. Table 2 summarizes the characteristics and function of apoVs in cancer.

**Table 2:** Characteristics and function of ApoVs in cancer

Origin	Inducing method	Centrifugal force	Size	Functional molecule	Biological effects	Terms used	References
Mice B16-F1 or B16-ovalbumin	Doxorubicin	100,000 × g for 60 min	357 ± 112.1 nm	Fibrin/PMEL/Thrombin	Procoagulant activity /immunogenic functions and the anti-cancer response	Apoptotic blebs or vesicles	[56]
Human and murine cancer cell lines	Doxorubicin	25,000 ×g for 60 min	103–816 nm 220–531 nm	Fibrin and thrombin generation	ApoV enhances procoagulant effects	Apoptotic vesicles	[57]
Human glycan-modified melanoma cell line Mel-JuSo	Bortezomib	10,000× *g*	100–1000 nm	Glycan	Antigen source for antitumor vaccination	Apoptotic extracellular vesicles	[58]
Mice bone marrow MSCs	Staurosporine and serum-free	16,000× *g*	50–250 nm	Fas ligand	Promoting Ca2 + influx and activating the Fas-FasL pathway, Inducing multiple myeloma cell cell apoptosis	Apoptotic extracellular vesicles	[54]
External tumor cells	Nanoparticles loaded with camptothecin	1000× *g*	1.2 μm	PR104A	Carry the remaining anti-tumor drugs to neighboring tumor cells	Apoptotic bodies	[59]
Mouse lymphoma cells	UV radiation	Sequential centrifugation	1–5 μm	Gold-silver nanorods (AuNR) -CpG	Preventing tumor metastasis and recurrence	Apoptotic body	[60]
RAW 264.7 cells and BMDCs	Fe and H2O2	Centrifugation		STING nanoparticles	Potentiate immunotherapy efficacy	Apoptotic Body	[61]
Lung adenocarcinoma cells	Cisplatin and Staurosporine	16,000× g for 30 min		ALDH1A1	Promote lung adenocarcinomametastasis		[3]

Interestingly, emerging evidence reveals significant functional of cancer stem cell (CSC)-derived exosomes and ApoEVs, particularly in their ability to remodel the TME and facilitate immune evasion. Although they arise from distinct biogenetic pathways, these vesicles often exhibit overlapping surface markers, such as phosphatidylserine exposure immunosuppressive ligands, oncogenic proteins, and metabolic enzymes [62]. For example, both vesicle subtypes have been shown to promote T cell exhaustion via the PD-1/PD-L1 axis and activate transcription (STAT) signaling [63]. Given the well-established role of CSC exosomes, insights from this field can substantially advance our understanding of ApoEV-mediated signaling. Specifically, mechanisms by which CSC exosomes transfer stemness-related factors, drive metabolic reprogramming, and confer therapy resistance offer a valuable conceptual framework for exploring analogous functions of ApoEVs. Integrating this cross-disciplinary knowledge may help uncover conserved principles underlying tumor-derived vesicle biology. Ultimately, such integration could accelerate the development of novel therapeutic strategies that concurrently target both CSC exosome and ApoEV pathways, thereby disrupting tumor progression and overcoming immunosuppression in the TME.

**Fig. 2:**
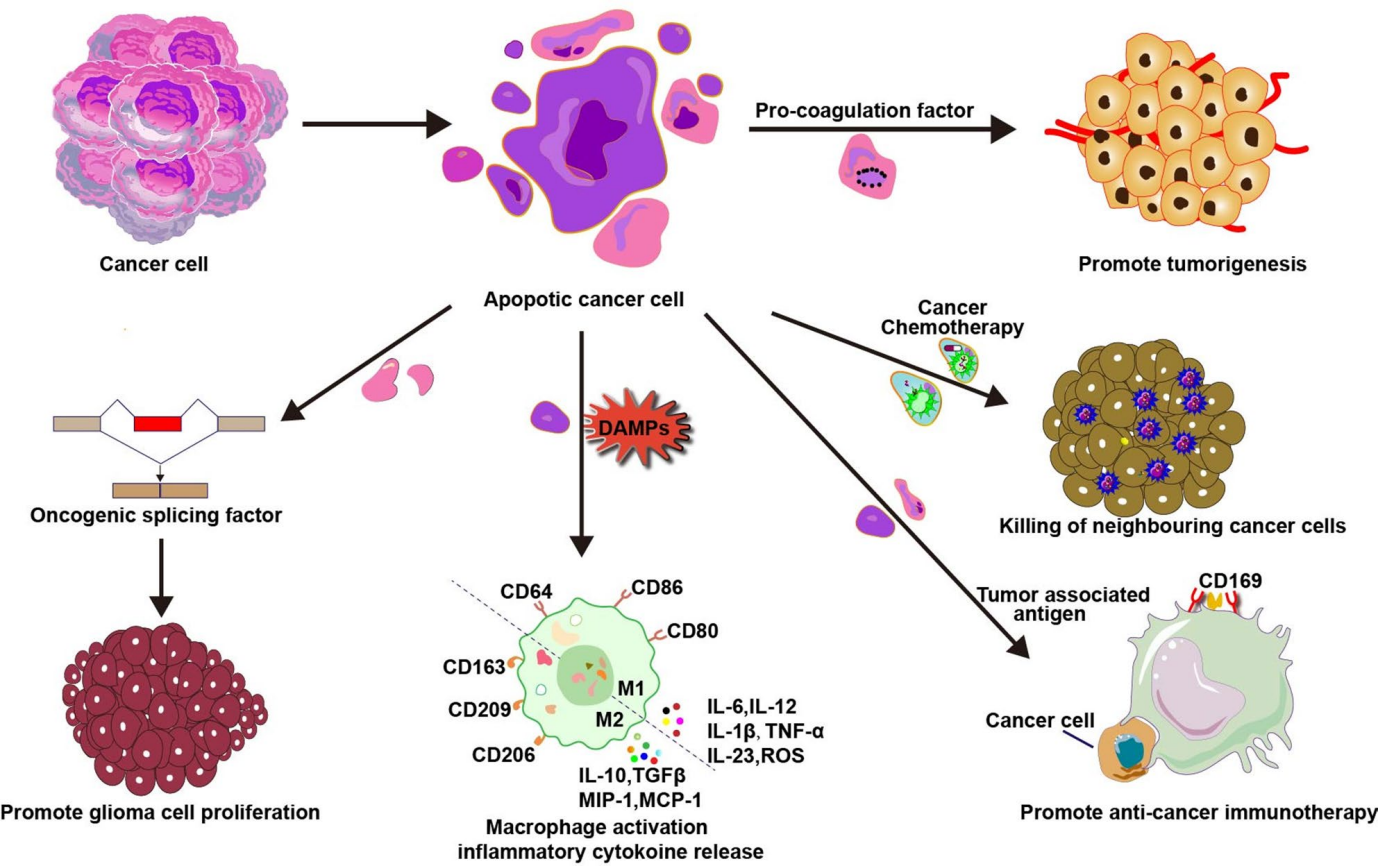
ApoEVs trigger multiple cellular responses in tumor cells, depending on their cellular origin and the biomolecules being transported

Taken together, ApoEVs constitute a microenvironment-dependent paradox in cancer (Fig. 2). They function as accomplices in tumor progression by delivering oncogenic cargo like RBM11/ALDH1A1, inducing EMT, promoting therapy resistance, and enabling immune evasion. Yet they can be weaponized against cancer, such as through MSC-ApoEV-induced apoptosis or melanoma ApoEV-enhanced immunosurveillance. This duality highlights the therapeutic potential of targeting ApoEV pathways, through ALDH1A1 inhibition or engineered immunomodulatory vesicles to combat tumor recurrence and drug resistance.

## Apoptotic vesicles for cancer therapy

### Apoptotic vesicles as vaccines

Cancer immunotherapy leverages enhanced immune responses, broad-spectrum antitumor effects, and durable remissions, driving interest in ApoEV vaccines. ApoEVs are promising antigen sources as they inherit tumor-associated antigens (e.g., PMEL) or pathogen components from parental cells, enabling efficient cross-presentation by macrophages and dendritic cells (DCs) to activate CD8⁺ T-cell immunity. Early evidence demonstrated that sodium butyrate-induced ApoEVs combined with IL-2 significantly improved survival and stimulated specific antibody production in colorectal cancer models [64]. Subsequent clinical advances further established that allogeneic DCs loaded with ApoEVs reduce tumor burden, elevate proinflammatory factors in chronic B-lymphocytic leukemia patients, and exhibit favorable tolerability [65].

In a clinical trial investigating early leukemia immunotherapy, allogeneic dendritic cells (DCs) loaded with tumor lysates or ApoBDs demonstrated efficacy and tolerability. Specifically, vaccination reduced peripheral leukocytes and CD19+/CD5 + leukemia cells while elevating pro-inflammatory cytokines (TNFα, IFNγ, IL-2) in most patients [66]. Furthermore, Palma et al. developed autologous leukemic apoptotic body-loaded DC vaccines (Apo-DC) for chronic lymphocytic leukemia patients, observing only mild injection-site reactions alongside confirmed safety [67]. Critically, these Apo-DC vaccines induced in vivo antitumor immunity, with adjuvants granulocyte-macrophage colony-stimulating factor (GM-CSF) and low-dose cyclophosphamide exerting additive immunomodulatory effects [67]. However, vaccine efficacy exhibits antigen-dependent variability. Notably, melanoma apoptotic vesicle-loaded DCs showed limited clinical efficiency requiring combinatorial therapies [68].

Engineering strategies have used to optimize ApoEV vaccine vectors. High-mannose glycan-modified ApoEVs enhance CD8⁺ T-cell activation by targeting DC C-type lectin receptors [58]. Percutaneously inoculated ApoEVs are efficiently internalized by dermal DC subsets to promote tumor antigen cross-presentation [69, 70]. Combination therapies demonstrate synergistic potential: apoptotic DC vaccines (Apo-DCs) with GM-CSF and cyclophosphamide amplify immune responses, whereas standalone melanoma ApoEV-DC vaccines require adjuvant therapies [67].

Despite these advances, translational challenges persist, including functional heterogeneity linked to vesicle size (e.g., IL-1α enrichment in 1–3 μm vesicles versus absence in < 1 μm subtypes), inconsistent terminology conflating ApoEVs with apoptotic debris, and contradictory reports regarding DC maturation induction. Moreover, the absence of standardized isolation protocols impedes systematic compositional analysis. Future progress hinges on elucidating ApoEV biogenesis and antigen distribution while establishing isolation criteria based on biophysical parameters. Crucially, the natural multi-antigen delivery capacity of ApoEVs combined with engineerable features (e.g., DC-targeting ligands) sustains their unique strategic value for next-generation vaccine development.

### Apoptotic vesicles as drug carriers

ApoEVs demonstrate significant advantages as drug carriers compared to artificial liposomes, leveraging natural cell-cell communication, target recognition, and biosignaling responsiveness. Notably, ApoEVs derived from apoptotic cells exhibit higher production efficiency than exosomes or microvesicles. Critically, apoptosis actively packages biomolecules into vesicles, enabling efficient drug loading and highlighting ApoEVs’ remarkable potential as antitumor carriers. However, ApoEVs exhibit dual therapeutic roles: while delivering chemotherapeutic agents to neighboring cells, they may inadvertently transport carcinogens or regulatory factors influencing tumor progression. For instance, paclitaxel/doxorubicin-loaded s-EVs induce cancer cell apoptosis [71], and engineered pre-drug-loaded cancer cell-derived ApoEVs eliminate adjacent malignant cells [59].

**Fig. 3:**
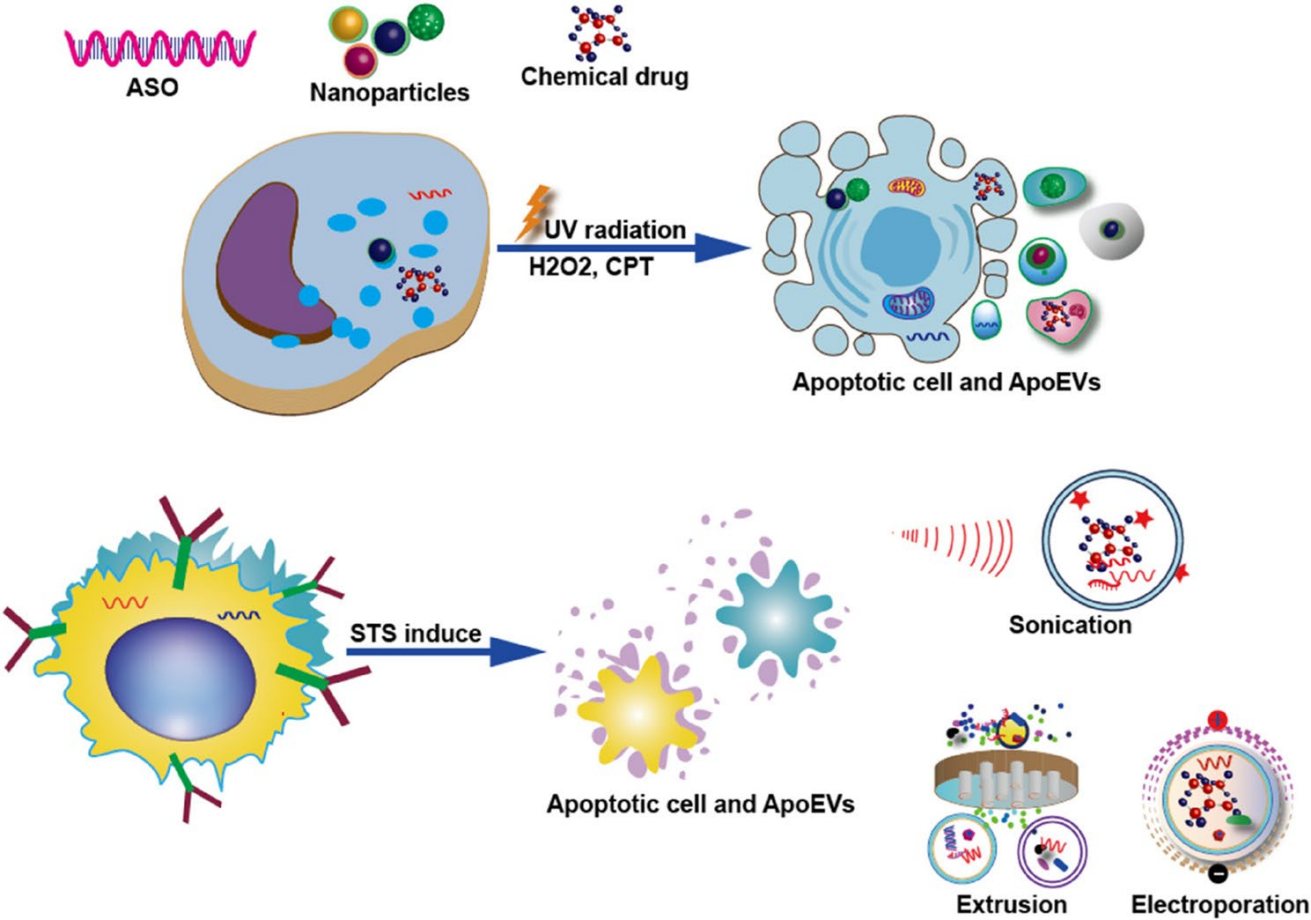
Methods for constructing ApoEVs as vectors. Multiple strategies have been developed for cargo loading, broadly categorized into passive and active approaches. Passive enrichment involves transfecting parental cells with nucleic acids, nanoparticles, or compounds prior to apoptosis induction (e.g., via UV/H₂O₂), whereby therapeutic payloads become encapsulated during ApoEV biogenesis. In contrast, active drug loading employs post-isolation techniques—including electroporation, sonication, saponin-mediated permeabilization, freeze-thaw cycling, and extrusion to directly incorporate cargo into purified ApoEVs

Nevertheless, cargo loading remains challenging when utilizing ApoEV delivery platforms. Two primary strategies exist: pre-loading cargo into parental cells prior to apoptosis induction, or post-loading into pre-formed ApoEVs (Fig. 3). The former approach is exemplified by Wang et al. [72], who transfected ASO into cells *via* cationic konjac glucomannan before UV/H₂O₂-induced apoptosis, generating CD44v6-regulated ApoEVs that crossed the blood-brain barrier to treat Parkinson’s disease. Similarly, Zheng et al. achieved blood-brain barrier penetration using AuNR-CpG-loaded tumor cells [73], while their photodynamic therapy study utilized 630 nm laser excitation of PpIX-loaded lymphocytes to generate therapeutic ApoEVs [46]. Conversely, Zhang et al. [7] employed post-loading by inducing 4T1 cell apoptosis *via* chemical membrane blistering/extrusion, then electroporation-loaded saporin/siRNA to form ABA complexes [74]. These complexes inherited CD47 proteins for anti-phagocytosis and homologous targeting, demonstrating potent tumor accumulation that suppressed proliferation and lung metastasis in 4T1 models.

Alternatively, Bose et al. [75] generated tumor-derived ApoEVs through starvation, then sonicated/freeze-thawed them with vancomycin before membrane extrusion. This recombination significantly reduced vesicle size and increased antibiotic encapsulation efficiency. Consequently, vancomycin-loaded ApoEVs targeted macrophages and tumor cells more effectively than free drug, eliminating intracellular *Staphylococcus aureus* in vitro/vivo. Meanwhile, Zuo et al. [76] constructed HAL/3BP@ApoEVs by X-ray-induced apoptosis followed by electroporation loading of photosensitizers (HAL/3BP), achieving synergistic tumor-targeted photodynamic therapy after intravenous administration.

**Fig. 4:**
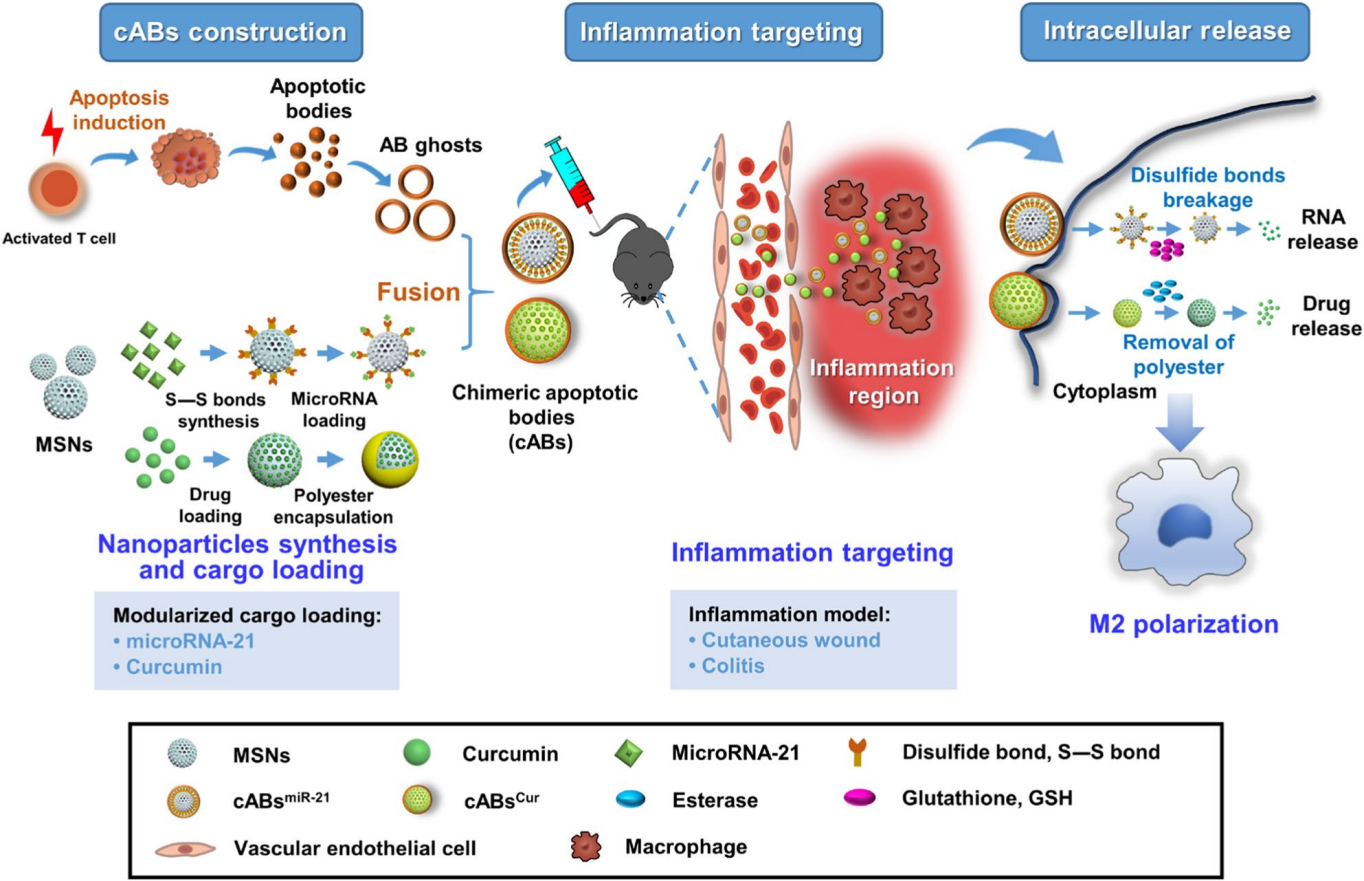
Schematic illustration of the cargo chimeric ApoEVs that were accurately released at a specified location by utilizing pure membranes of ApoEVs as bioconjugate/regulatory modules and mesoporous silica nanoparticles (MSNs) as carrier modules. Reproduced from ref. [38] © 2025 American Association for the Advancement of Science

Fundamentally, phosphatidylserine (PS) on ApoEV surfaces serves as an “eat-me” signal for monocyte/macrophage recognition, enabling targeted delivery. Accordingly, Dou et al. [38] engineered CABs using T-cell ApoEV membranes coating mesoporous silica nanoparticles, achieving stimulus-responsive release of cargo at locally target tissues (Fig. 4). Similarly, Zhang et al. developed PS-modified liposomes depleting tumor-associated macrophages (TAMs) to enhance efficacy [77]. Notably, ApoEV-mimicking liposomes deliver siRNA to macrophages for viral vector-free gene knockdown [78]. Innovatively, a two-step platform exploits tumor-derived ApoEVs to hijack monocyte homing for solid tumor accumulation [3].

Compared with extensively studied exosome-based drug carriers, ApoEVs similarly exhibit advantageous biological characteristics. However, scaling up ApoEV production presents greater technical challenges than for exosomes, because their yield depends heavily on the efficiency of apoptosis induction, and batch-to-batch heterogeneity tends to be more pronounced. In contrast, synthetic nanoparticles (e.g., liposomes, lipid nanoparticles) already benefit from multiple commercially available products and well-established manufacturing processes that are scalable, reproducible, and GMP-compliant. Such synthetic systems offer additional advantages, including chemically defined compositions, precisely controllable drug loading, and validated scale-up pathways. Nevertheless, they continue to face challenges such as “protein corona” formation and potential immunogenicity. Therefore, a promising future direction involves adapting existing liposome engineering expertise to develop “hybrid” or “biomimetic” carriers based on ApoEV membrane components. Such an approach could improve production consistency and scalability while retaining the essential biological functions of natural ApoEVs for drug delivery.

### Diagnostic role of apoevs in cancer

EVs serve as crucial intercellular mediators enriched with diagnostic signaling molecules from parental tumor cells. As core “liquid biopsy” tools, EVs demonstrate high stability, non-invasiveness, and dynamic monitoring capabilities, thus enabling widespread application in diagnosing cancers, cardiovascular disorders, and neurodegenerative diseases, as well as prognostic prediction. Similarly, cancer cell-derived ApoEVs represent novel liquid biopsy targets containing comprehensive tumor bioinformation that enables early diagnosis and prognostic evaluation.

Notably, Eerola et al. first reported apoptotic bodies’ diagnostic role in macrophages, observing that alveolar macrophages containing ApoEVs in lung cancer patients’ sputum smears provide more sensitive malignancy markers than malignant cells themselves, highlighting their potential for lung cancer detection [3]. Furthermore, apoptosis occurs ubiquitously in endocervical endometriosis, with apoptotic body counts directly correlating with mitotic division frequency. This correlation demonstrates ApoEVs’ association with endocervical adenocarcinoma in situ, suggesting their utility as supplementary grading variables [79]. Consistently, bladder cancer studies reveal apoptotic indices correlating with mitotic indices, tumor grade, and DNA ploidy. Specifically, Brustmann et al. found ApoEVs positively correlated with higher nuclear grades, mitotic activity, and aggressive growth patterns in plasmacytoid ovarian cancers [80], while Aihara et al. observed significantly increased apoptotic vesicles in prostate lesions versus non-tumor tissues, with ApoEVs abundance correlating positively with cancer grade [81]. Building on this prognostic value, Aydin et al. advocate including ApoEVs among histologic features for challenging prostate cancer needle biopsies [82].

Moreover, blood-derived ApoEVs extraction enables efficient, minimally-invasive liquid biopsy for disease monitoring [83]. However, ApoEVs heterogeneity complicates molecular characterization, as individual vesicle profiles may not reflect overall EV biology. Critically, tumor-derived ApoEVs molecular signatures dynamically evolve during cancer progression, challenging diagnostic marker stability and reliability. Therefore, establishing comprehensive standardized characterization systems, incorporating particle size, density, morphology, surface markers, and internal biomolecules is paramount.

ApoEVs demonstrate multifaceted therapeutic potential in oncology, emerging as a research hotspot for cancer immunotherapy and targeted drug delivery. This unique advantage lies in functioning simultaneously as natural immunomodulators, tumor antigen reservoirs, and drug delivery carriers, thereby enabling multi-pronged antitumor effects. Moreover, ApoEVs exhibit distinctive value in tumor microenvironment regulation. Specifically, tumor-associated macrophages (TAMs), key components of this microenvironment, typically display pro-tumorigenic M2 polarization [73]. Notably, engineered ApoEVs can reprogram TAMs toward tumoricidal M1 phenotypes and enhance their antitumor activity, significantly improving therapeutic efficacy [84]. For example, whereas hepatocellular carcinoma-derived EVs drive macrophage polarization toward M2 states, designed ApoEVs reverse this process and remodel immunosuppressive microenvironments into antitumor milieus.

Compared with conventional and emerging diagnostic biomarkers, ApoEVs possess distinct characteristics and potential advantages. Specifically, ApoEVs are selectively released during programmed cell death and carry molecular cargo that directly mirrors the physiological or pathological state of their parent cells. Moreover, ApoEVs encapsulate DNA within a protective phospholipid bilayer, which shields genetic material from rapid degradation by circulating nucleases. This protective feature allows ApoEVs to preserve disease-specific genetic and epigenetic information more effectively than cell-free DNA (cfDNA) alone. Additionally, because apoptosis often occurs early in disease progression, ApoEVs may enter circulation sooner than biomarkers that depend on substantial tissue damage or the late-stage release of proteins or abundant cfDNA.

## Conclusion and further perspectives

This review synthesizes the impact of ApoEVs on tumor progression, highlights their diagnostic and prognostic roles across malignancies, and illustrates therapeutic applications of ApoEV-associated proteins. It seeks to establish a comprehensive understanding of ApoEV biological mechanisms and clinical value, thereby advancing insights into their functions in tumorigenesis while guiding future research directions. To address inherent limitations in targeting and therapeutic efficacy, multiple engineered modifications have emerged including content modification via therapeutic molecule loading (miRNA/siRNA) using electroporation, sonication, or chemical transfection; surface engineering through genetic manipulation or chemical conjugation to display targeting ligands; pre-conditioning strategies altering ApoEV composition via hypoxic/pharmacological stimulation of parental cells and hybrid systems combining ApoEVs with synthetic materials for enhanced biomimetic delivery [85–87].

Despite these promising prospects, the clinical translation of engineered ApoEVs faces several critical challenges. These primarily concern scalable production, cargo safety, immunological risks, as well as standardization and regulatory pathways.

### Challenges in scalable production of ApoEV

Current ApoEV production largely depends on laboratory-scale cell culture and apoptosis induction, which is limited by low yield and significant batch-to-batch heterogeneity. ApoEV inherently exhibit variability in size and composition, hindering the ability to meet the bulk, uniform requirements for clinical-grade therapeutics. Future development must focus on scalable biomanufacturing platforms. This entails optimizing physical or biological apoptosis induction methods, utilizing bioreactors for large-scale synchronized culture and induction, and integrating novel separation techniques such as microfluidics. These approaches are essential for producing high-purity, high-yield ApoEVs with consistent characteristics, a challenge that is not only technical but also critical for treatment cost-effectiveness. 

### Potential risks of cargo safety

ApoEVs inherit and enrich biomolecules from their parent cells. When derived from tumor cells, they may carry oncogenic nucleic acids (e.g., mutant oncogenes, pro-metastatic miRNAs), abnormal signaling proteins, or residual drug precursors. Delivering such "harmful cargo" to recipient cells risks promoting tumor progression or inducing unintended toxicity. Even ApoEVs engineered to carry therapeutic molecules require rigorous evaluation of dose control, in vivo release kinetics, and off-target effects. Therefore, ensuring cargo safety necessitates stringent donor cell selection, developing purification processes that remove or inactivate harmful components, and implementing deep molecular profiling and functional safety testing of final products.

### Immunological and long-term safety considerations

The broadly exposed phosphatidylserine on ApoEV surfaces acts as an “eat-me” signal, promoting clearance by immune cells. However, PS may also mediate immunosuppression or inflammatory responses, with effects depending on the microenvironment. Furthermore, major histocompatibility complex molecules retained on ApoEVs from allogeneic cells could trigger immune rejection or autoimmune responses. Although ApoEVs generally exhibit lower immunogenicity than live cells, their long-term in vivo fate, accumulation potential, and chronic immunomodulatory effects remain unclear. Systematic preclinical toxicology studies to assesse immunogenicity, tumorigenicity, and biodistribution are therefore indispensable for clinical advancement.

### Standardization and GMP Compliance

The ApoEV research field currently lacks unified standards for isolation, characterization, and quality control. Significant variations in preparation methods, purity criteria, and functional descriptions of “ApoEVs” across studies severely hinder comparability and translation. Future translation must adhere to Good Manufacturing Practice (GMP) standards, requiring comprehensive standard operating procedures (SOPs) covering donor cell banking, production process control, and final product release testing. Critical Quality Attributes (CQAs) should include particle concentration, size distribution, marker expression (e.g., PS positivity), purity (freedom from cellular debris), potency (e.g., drug loading, targeting efficiency), and sterility. While the MISEV guidelines from the International Society for Extracellular Vesicles (ISEV) provide a characterization framework, specific industry standards and regulatory guidance for ApoEV therapeutics still require collaborative development among academia, industry, and agencies such as the FDA.

### Summary and outlook

In summary, transforming ApoEVs from promising research tools into reliable clinical therapies requires systematically addressing challenges in production, safety, quality control, and regulation. Future efforts should focus on developing scalable GMP-compliant production processes, elucidating and mitigating potential oncogenic and immunological risks, and establishing international consensus on standards and regulatory pathways. Overcoming these translational barriers is essential for ApoEVs to realize their full potential as next-generation delivery platforms and immunomodulators, ultimately benefiting cancer patients.

## Data Availability

Not applicable.
